# A Case of Severe Injury—Recovery

**Published:** 1865-08

**Authors:** O. D. Norton

**Affiliations:** Act’g. Ass’t. Surgeon, U. S. A.


### A Case of Severe Injury—Recovery.

TREATED BY O. D. NORTON, ACT'G. ASS'T. SURGEON, U. S. A.

Rev. William F. Nelson, aged 56, Chaplain, Washington Park, U. S. A. General Hospital, Cincinnati. On the 28th day of January, 1865, while in the discharge of his official duties he was knocked down and stunned by the pole of a two-horse sleigh. While on the ground, the horse's foot struck him in the face, over the right molar bone, fracturing and separating the entire upper jaw from its attachments. The external wound, commencing at the tuberosity of the right molar bone, extended horizontally across the nose, immediately beneath the external canthus of the right eye, then vertically downwards to the right angle of the mouth.

Commencing upwards and going downwards, we find the following injuries: first, a fracture of the several bones composing the floor of the right eye; next, a comminuted fracture of both nasal bones, and their separation from the attachment to the frontal bone. The right superior maxillary bone was also fractured, it running through the antrum hymorianum. Both maxillary bones were loosened from their attachments of the pterygoid plate of the sphenoid. The whole of the right side of the maxillary bone was thrown forward in such a manner, that in looking below, the fauces and epiglottis were plainly visible. The palate bones were separated from one another, and portions of the soft palate torn and lacerated. The last two molars of the upper jaw, and also of the lower, on the right side, and last molar of lower jaw on the left side were loosened. A rib was also fractured on the right side. * * * * * * *

The health of the patient has now been fully restored, although he experiences some loss of the power of mastication and speech. The latter faculty seems to be improving, and the patient expresses an earnest trust that, although he will never be able to articulate as well as he could formerly, it will continue to improve. The lacrymal ducts have been obliterated. At the present writing, there exists still more looseness of the superior maxillary bone. In all other respects the patient is unusually well.—*Cincinnati Lancet and Observer.*

